# Dry Needling for Patients With Neck Pain: Protocol of a Randomized Clinical Trial

**DOI:** 10.2196/resprot.7980

**Published:** 2017-11-22

**Authors:** Eric Robert Gattie, Joshua A Cleland, Suzanne J Snodgrass

**Affiliations:** ^1^ Rehabilitation Services Concord Hospital Concord, NH United States; ^2^ Physical Therapy Program Franklin Pierce University Manchester, NH United States; ^3^ University of Newcastle Callaghan Australia

**Keywords:** neck pain, dry needling, physical therapy

## Abstract

**Background:**

Neck pain is a costly and common problem. Current treatments are not adequately effective for a large proportion of patients who continue to experience recurrent pain. Therefore, new treatment strategies should be investigated in an attempt to reduce the disability and high costs associated with neck pain. Dry needling is a technique in which a fine needle is used to penetrate the skin, subcutaneous tissues, and muscle with the intent to mechanically disrupt tissue without the use of an anesthetic. Dry needling is emerging as a treatment modality that is widely used clinically to address a variety of musculoskeletal conditions. Recent studies of dry needling in mechanical neck pain suggest potential benefits, but do not utilize methods typical to clinical practice and lack long-term follow-up. Therefore, a clinical trial with realistic treatment time frames and methods consistent with clinical practice is needed to examine the effectiveness of dry needling on reducing pain and enhancing function in patients presenting to physical therapy with mechanical neck pain.

**Objective:**

The aim of this trial will be to examine the short- and long-term effectiveness of dry needling delivered by a physical therapist on pain, disability, and patient-perceived improvements in patients with mechanical neck pain.

**Methods:**

We will conduct a randomized, double-blind, placebo-controlled trial in accordance with the CONSORT guidelines. A total of 76 patients over the age of 18 with acute or chronic mechanical neck pain resulting from postural dysfunction, trauma, or insidious onset who are referred to physical therapy will be enrolled after meeting the eligibility criteria. Subjects will be excluded if they have previous history of surgery, whiplash in the last 6 weeks, nerve root compression, red flags, or contraindications to dry needling or manual therapy. Participants will be randomized to receive (1) dry needling, manual therapy, and exercise or (2) sham dry needling, manual therapy, and exercise. Participants will receive seven physical therapy treatments lasting 45 minutes each over a maximum of 4 weeks. The primary outcome will be disability as measured by the Neck Disability Index. Secondary outcomes include the following: pain, patient-perceived improvement, patient expectations, and successful blinding to the needling intervention. Outcome measures will be assessed at 4 weeks, 6 months, and 12 months by an assessor who is blind to the group allocation of the participants to determine the short- and long-term treatment effects. We will examine the primary aim with a two-way, repeated-measures analysis of variance with treatment group as the between-subjects variable and time as the within-subjects variable. The hypothesis of interest will be the two-way group by time interaction. An a priori alpha level of .05 will be used for all analyses.

**Results:**

Recruitment is currently underway and is expected to be completed by the end of 2017. Data collection for long-term outcomes will occur throughout 2017 and 2018. Data analysis, preparation, and publication submission is expected to occur throughout the final three quarters of 2018.

**Conclusions:**

The successful completion of this trial will provide evidence to demonstrate whether dry needling is effective for the management of mechanical neck pain when used in a combined treatment approach, as is the common clinical practice.

**Trial Registration:**

ClinicalTrials.gov NCT02731014; https://clinicaltrials.gov/ct2/show/NCT02731014 (Archived by WebCite at http://www.webcitation.org/6ujZgbhsq)

## Introduction

Neck pain is common, with 30%-50% of the population afflicted in a given year [1]. Symptoms of neck pain persist longer than 12 months in 37% of patients [2]. Neck pain is ranked fourth highest out of all 291 conditions studied in the Global Burden of Disease 2010 study measured by years lived with disability [3]. In the United States, estimated increases in expenditures for patients with spine pain have increased 65% from 1997 to 2005 [4]. Patients with neck pain account for 10%-20% of all patients seen in outpatient physical therapy [5,6].

Commonly, physical therapists utilize patient education, exercise, mobilization, manipulation, massage, and electrophysical modalities when treating patients with mechanical neck pain. Yet the most recent Cochrane reviews on patient education [7], exercise [8], and manual therapy (ie, joint mobilization [9] and massage [10]) find a lack of high-quality evidence to support these interventions independently. There is some evidence that multi-modal physical therapy treatment consisting of a combination of exercise and mobilization/manipulation seems to be more effective than either intervention alone [11].

Dry needling has emerged as a treatment modality that is widely used in the clinical environment to address a variety of musculoskeletal conditions including neck pain [12-14]. Dry needling is growing in popularity despite a lack of clinical trials examining its effectiveness, likely due to the ease of applying dry needling in a clinical setting [15,16]. Dry needling is a technique in which a fine needle is used to penetrate the skin, subcutaneous tissues, and muscle with the intent to mechanically disrupt tissue without the use of an anesthetic [17].

Most commonly, dry needling targets myofascial trigger points (MTrPs), which are described as localized hypersensitive spots in a palpable taut band of muscle [18-28]. These hyperirritable spots can be classified as active MTrPs when they produce spontaneous pain and, when palpated, reproduce a patient’s familiar pain. Latent MTrPs do not produce spontaneous pain and are only painful upon palpation [29]. Many studies have shown that MTrPs are prevalent in patients with chronic neck pain [30-34]. It has been reported that MTrPs in the neck and shoulder commonly result in limited range of motion in the neck, neck pain, headache, and dizziness [28,33,35].

There have been five recent studies examining dry needling performed by a physical therapist for patients with neck pain. Four of the trials examined the short-term effectiveness of dry needling: three in chronic mechanical neck pain [21,24,28] and one in acute mechanical neck pain [23]. The results of these studies demonstrated that dry needling decreases pain intensity and increases pain pressure threshold in the short term; the longest follow-up was 4 weeks. One recent trial examined the long-term effectiveness of dry needling, which was performed on patients with chronic nonspecific neck pain [36]. At 6-month follow-up, the dry needling and passive stretching group demonstrated significant and clinically relevant improvements in pain, disability, and range of motion when compared to passive stretching alone. These results suggest that dry needling warrants further investigation for the treatment of neck pain.

Recent systematic reviews suggest that dry needling can be recommended in the short and medium term to reduce neck and shoulder pain [37] and for musculoskeletal pain [38]. Yet those reviews concluded that there was limited evidence to support dry needling’s effectiveness in the long term for reducing pain or improving function, especially when compared to other physical therapy interventions. Both authors recommended further studies with adequate sample sizes to examine dry needling effectiveness in both the short and long term on reducing pain and improving function.

The majority of the studies included in the recent reviews compared dry needling to control, sham, or to another intervention directly. Not only is this not consistent with how dry needling is commonly used clinically by physical therapists, but emerging evidence suggests that dry needling, when performed in combination with other interventions, may be more effective at reducing lower back pain than when performed alone [39]. This suggests that dry needling, when combined with other interventions (ie, multi-modal treatment), may result in an improved treatment effect when compared to dry needling performed in isolation.

The majority of existing studies lack adequate sample sizes, only collect short-term outcomes, frequently examine dry needling as a stand-alone intervention, and only treat one muscle for one to two sessions, which is not consistent with how dry needling is commonly performed clinically. Typically, clinicians will perform an examination to locate active and latent MTrPs and needle numerous active trigger points in a single session. They may then dry needle the patient over many sessions to achieve optimal effects. With the high prevalence of neck pain and its contribution to prolonged disability in patients, it is essential to identify optimal treatment approaches [1-3,5,6,40,41]. Therefore, the aim of this randomized clinical trial is to examine the long-term effects of a combination of manual therapy, exercises, and dry needling to the cervicothoracic region on pain and disability in individuals with acute or chronic mechanical neck pain resulting from postural dysfunction, trauma, or insidious onset who are referred to physical therapy.

We hypothesize that patients who receive dry needling, manual therapy, and exercise will achieve greater reductions in pain and disability in the short term (ie, 4 weeks) and long term (ie, 6 and 12 months) compared to those who receive sham dry needling, manual therapy, and exercise.

## Methods

### Design

**Figure 1:**
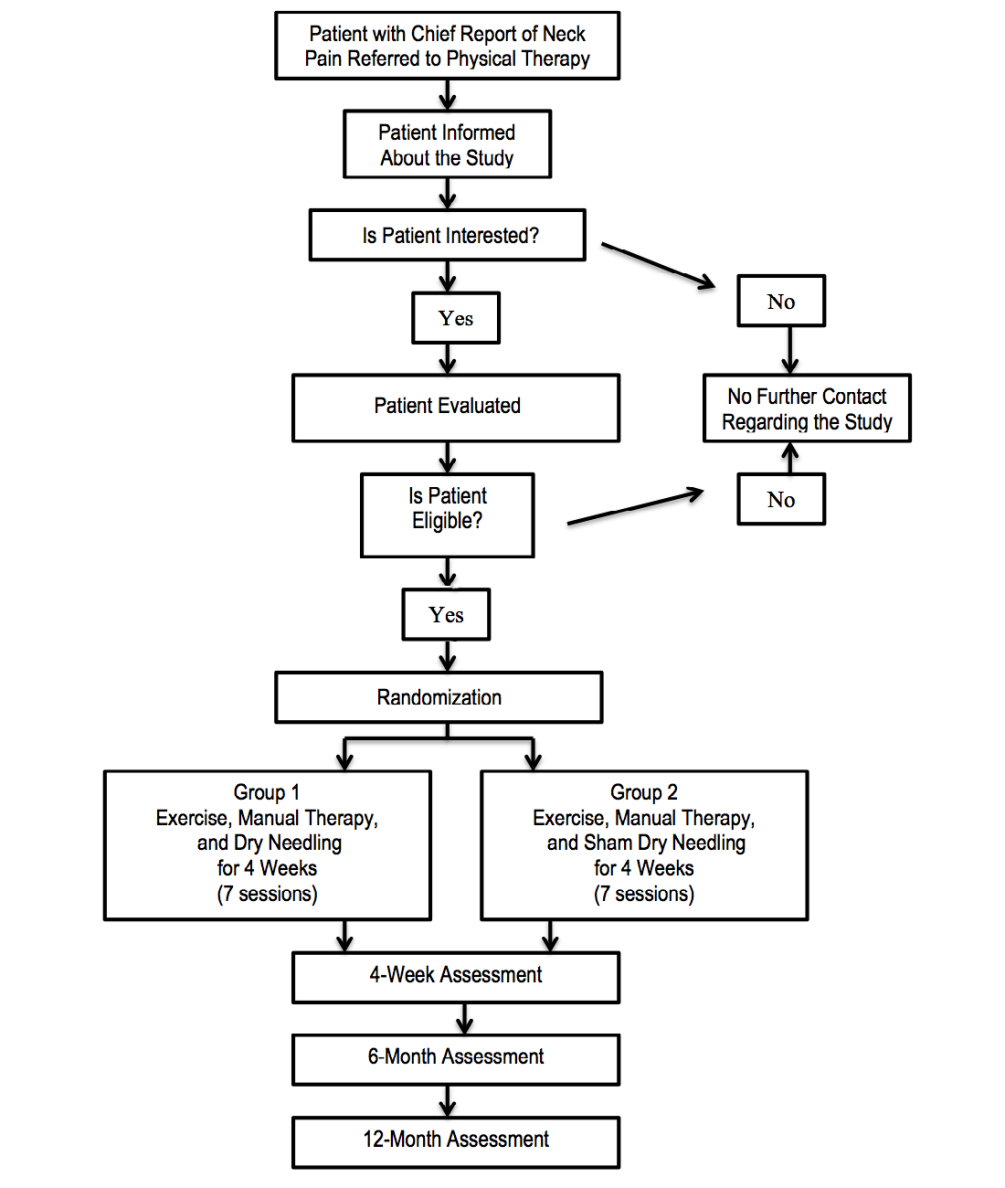
Flowchart of the study trial.

We will conduct a randomized, double-blind, placebo-controlled trial according to the CONSORT guidelines (see Figure 1) [42,43]. Approval by both the Institutional Review Board at Concord Hospital (Concord, NH, USA), where the trial will be performed, and the University of Newcastle Human Research Ethics Committee (Callaghan, Australia), where the primary author (ERG) is currently enrolled as a PhD candidate, have been obtained. Consecutive subjects presenting to Concord Hospital physical therapy clinics (Concord, NH, USA) and Franciscan St. Francis Health physical therapy clinics (Indianapolis, IN, USA) with mechanical neck pain will be screened for eligibility criteria.

### Subjects

Each physical therapy clinic, upon receiving a patient referral for neck pain, attempts to schedule the patient with the therapists who have agreed to assess and treat participants in the trial. Upon arrival for their initial assessments, patients are informed about the potential opportunity to participate in the study if they meet inclusion/exclusion criteria. A combination of physical examination and self-report measures will be used to assess each patient’s potential eligibility to participate. Inclusion criteria for the study include the following: aged 18 years or older, a primary complaint of neck pain, and a Neck Disability Index (NDI)>10 points=20% [44]. Patient exclusion criteria include the following: red flags (ie, tumor, fracture, metabolic diseases, rheumatoid arthritis, osteoporosis, prolonged history of steroid use, symptoms of vertebrobasilary insufficiency, pregnancy, cervical spinal stenosis, or bilateral upper extremity symptoms); use of blood thinners; history of whiplash injury within the past 6 weeks; evidence of central nervous system involvement, including hyperreflexia, sensory disturbances in the hand, intrinsic muscle wasting of the hands, unsteadiness during walking, nystagmus, loss of visual acuity, impaired sensation of the face, altered taste, or the presence of pathological reflexes such as positive Hoffman’s and/or Babinski reflexes; two or more positive neurologic signs consistent with nerve root compression (ie, muscle weakness involving a major muscle group of the upper extremity, diminished upper extremity muscle stretch reflex, or diminished or absent sensation to pinprick in any upper extremity dermatome); prior surgery to the neck or thoracic spine; workers’ compensation or pending legal action regarding their neck pain; insufficient English-language skills to complete all questionnaires; and inability to comply with treatment and follow-up schedule.

If a patient is determined to have met inclusion/exclusion criteria, they will be asked if they would like to participate in the study. If they choose to provide informed consent, each patient will complete the baseline questionnaires as well as the baseline outcome assessments. Demographic information will be collected for descriptive purposes, including age, gender, employment status, past medical history, mechanism of injury, location and nature of the patient’s symptoms, number of days since onset, number of previous episodes of neck pain, and treatment for previous episodes. Once patients are admitted to the study, no patient will be removed from the study for noncompliance and an intention-to-treat analysis will be used.

### Outcome Measures

#### Overview

All subjects will complete several commonly used instruments to assess their level of disability and neck pain. The primary outcome measure will be the NDI to capture the effect of treatment on the level of disability. Secondary outcome measures will include the Visual Analog Scale (VAS) to assess pain, the Global Rating of Change Scale (GROC) to measure the patient’s perceived recovery, the patient perception of the intervention to determine if they felt they received the genuine dry needling intervention, and patient expectations to determine which treatments they believed would be most beneficial for their current condition. The self-report measures that will be used include those discussed in the following sections.

#### Primary Outcome

The NDI was created to measure pain-related disability associated with activities of daily living in people with neck pain [45,46]. The NDI contains 10 focused sections. Each item is scored on a 6-point scale and can reach a maximum score of 5; therefore, the maximum score is 50. This score will be calculated as a percentage, with higher scores indicating higher levels of disability [46]. Content, construct validity, and reliability of the NDI has been previously shown in patients with neck pain [46,47]. The NDI has been used by researchers to evaluate the effect of treatments on patients’ perceived levels of functioning and disability [48-51].

#### Secondary Outcomes

##### Visual Analog Scale

The VAS is a single-item measure of pain using a 100-mm horizontal line anchored on the left side, which represents *no pain*, while the right side represents *the worst pain imaginable*. Patients mark a score by making a vertical line where they feel best represents their pain intensity. The VAS has been shown to be reliable [52] and valid [53].

##### Patient Global Rating of Change

The GROC will be used, which is a 15-point global rating scale described by Jaeschke et al [54]. The scale ranges from –7 (*a very great deal worse*) to zero (*about the same*) to +7 (*a very great deal better*). The global rating will be administered at the follow-up examinations only.

##### Patient Perceptions

Patient perceptions about the intervention will be used to determine whether the sham was an effective placebo. Patients will be asked for their perceptions on the dry needling intervention they received in order to determine if they felt they received genuine treatment. The following questions will be asked: What did you think of the dry needling intervention? Would you be willing to have the dry needling intervention again if you were attending physical therapy? Do you think you received the real dry needling intervention?

##### Patient Expectations

Patient expectations will be assessed at enrollment into the trial. Patients will be asked to rate each intervention on a 5-point Likert scale as to whether they believe each specific intervention will significantly help to improve this episode of their neck pain.

All outcome assessments will be performed by an individual blind to group assignment and will be performed at baseline, 4 weeks, 6 months, and 12 months. The latter two assessments will be mailed to the patients to improve return rate (see Table 1) [55].

**Table 1:** Summary of outcome measures and time points for collection.

Outcome measure	Baseline	4 weeks	6 months	12 months
Neck Disability Index	Yes	Yes	Yes	Yes
Visual Analog Scale	Yes	Yes	Yes	Yes
Global Rating of Change	No	Yes	Yes	Yes
Patient perception of intervention	No	Yes	Yes	Yes
Patient expectations	Yes	No	No	No

### Intervention

#### Overview

Once the baseline examination is complete, equal numbers of patients will be randomly assigned to one of two groups: (1) manual therapy, exercise, and dry needling (MTEX-Needle group) or (2) manual therapy, exercise, and sham dry needling (MTEX-Sham group). A random-number generator will be used to conduct the randomization. The randomization procedure will be conducted prior to the initiation of the study using a computer program randomizer by an individual not involved in patient recruitment. The randomization will be concealed according to the following procedure. The group assignment will be recorded on a label that will be placed inside an envelope and the envelope will be sealed. After the baseline examination is complete, the randomization envelope will be handed to a treating therapist and treatment will begin according to group assignment. Treatment will be initiated immediately following the baseline examination, unless prohibited by time constraints; in this case, the patient will be scheduled for a follow-up session within 3-5 days to receive the first treatment. Patients in both groups will attend physical therapy for seven treatments over a maximum of 4 weeks. Each treatment session will last for a total of 45 minutes of one-on-one treatment time with the treating physical therapist.

To ensure that the clinicians involved in administering the treatment are familiar with the procedures of the study, they will be required to participate in a 2-hour training session. During the training session, the manual therapy, exercise, and dry needling techniques will be reviewed to ensure treatments are applied in a standardized manner consistent with the treatment algorithm outlined below. The majority of the training time (ie, 1 hour) will be dedicated to ensuring standardization of the application of the dry needling and sham needling techniques. The second hour will be spent reviewing the manual therapy techniques, therapeutic exercises, and algorithms to help standardize their prescription, as well as data collection procedures. Due to the pragmatic nature of this study design, even when the treatment algorithm is followed for manual therapy and exercise, there will likely be some variation in the interventions selected by each therapist based on each patient’s relevant examination findings. As this is a pragmatic trial designed to mimic usual clinical practice, this individualization of a patient’s treatment is acceptable and expected. All therapists applying all interventions will be licensed physical therapists who have also completed the required postgraduate training that enables them by their state practice act to utilize dry needling, and who regularly use the technique in practice.

#### Manual Therapy: 15 Minutes

Individuals randomized to both groups will receive manual therapy to address joint mobility of the cervical and thoracic spine. Mobilization of the cervical spine and thrust manipulation targeting the thoracic spine will occur at the beginning of each treatment. The treatment algorithm, combined with physical examination findings, will guide clinicians to allow them to determine which techniques will be used and is outlined below (see Table 2).

#### Physical Examination Techniques That Will Guide Manual Therapy Intervention

##### Cervical/Thoracic Spine Active Range of Motion and Behavior of Symptoms

The examiner will record a single range of motion measurement for flexion and extension using an inclinometer as described by Hole [56]. Bilateral rotation will be measured using a standard long-arm goniometer [57]. Reliability coefficients for cervical spine range of motion parameters range from .81-.84 (inter-class correlation [ICC] 2,1) [56]. Thoracic rotation active range of motion will be assessed qualitatively. Patients will be asked to place their hands on opposite shoulders and to rotate the trunk. Care will be taken to maintain the cervical spine in neutral while the patient rotates the trunk to the left and right as far as possible. The behavior of symptoms and the presence of side-to-side asymmetry will be recorded.

##### Spring Testing

Spring testing of the cervical and thoracic spine over the spinous processes of the vertebrae will be tested with the patient prone [58,59]. The stiffness at each segment will be judged as normal, hypomobile, or hypermobile. Interpretation of whether a segment is hypomobile will be based on the examiner’s anticipation of what normal mobility should feel like at that level and compared to the mobility detected in the segment above and below. In addition, pain provocation at each segment will be judged as painful or not painful and, if painful, whether the symptoms are local (ie, under the examiner’s hand) or referred (ie, away from the examiner’s hand). Spring testing for the neck will be performed over the spinous processes of C2-C7. Spring testing for the thoracic spine will be performed over the spinous processes of T1-T5. The reliability of spring testing from our previous work showed poor reliability in the cervical spine and fair-to-moderate reliability in the thoracic spine [60].

**Table 2:** Manual intervention algorithm for treatment selection.

Assessment	Treatment
Clinicians assess cervical spine mobility and range of motion, including overpressure and repeated movements, if indicated	If hypomobility or limited range of motion is identified in the cervical spine, the therapist will utilize cervical thrust manipulation or nonthrust mobilizations; this may include central and unilateral posterior-anterior, side glides, and occipito-atlanto joint (C0-1)
	Thrust manipulations may be repeated up to two times if reassessment of the patient shows improvements in range of motion, mobility, and/or pain
	Nonthrust mobilizations generally performed two to three times ×30 repetitions at each hypomobile level and may be repeated again (two to three times ×30 repetitions) if the patient shows improvements in range of motion, mobility, and/or pain
Clinicians assess thoracic spine mobility and range of motion	If hypomobility or limited range of motion is identified in the thoracic spine, the therapist will utilize thoracic thrust manipulation and/or nonthrust manipulation (may include central and unilateral posterior-anterior techniques to the thoracic spine and ribs)
	Thrust manipulation will be used unless contraindications noted (history or self-report of osteopenia/osteoporosis, etc)
	Thrust manipulations may be repeated up to two times if reassessment of the patient shows improvements in range of motion, mobility, and/or pain
	Nonthrust manipulations generally performed two to three times ×30 repetitions at each hypomobile level and may be repeated again (two to three times ×30 repetitions) if the patient shows improvements in range of motion, mobility, and/or pain

**Table 3:** Exercise intervention algorithm for treatment selection.

Assessment	Treatment	Progression
Muscular endurance of the cervical flexors was evaluated with the deep neck flexor endurance test and evaluated based on hold time in seconds	Prepare participant in supine, hook-lying position and ensure craniocervical and cervical regions are in a neutral position (support with a folded towel if necessary). Teach the craniocervical flexion action. Use instructions of “feel the back of your head slide up the bed as you nod your chin.” Goal: 10 × 10-second holds	Begin with craniocervical flexion. Cue patient to “keep chin tucked in, lift, and hold your head up.” Goal: 10 × 10-second holds
Craniocervical and cervical extensors	Patient either prone on elbows or in four-point kneeling position. Suboccipital muscles—Focus on a neutral neck position: (1) Require the participant to perform craniocervical extension (chin down) and (2) Require the participant to perform craniocervical rotation (the saying “no” action). Assess quality of movement and for smooth coordination. Goal: 3 sets of 5	Patient either prone on elbows or in four-point kneeling position. Deep cervical extensors: the craniocervical region remains in neutral and the axis of motion is now at C7. Instruct the participant to curl their neck first into flexion and then to curl their neck back to extension. The participant will often require manual facilitation to achieve the correct action. To assist in maintaining the craniocervical neutral position, let the participant imagine they have a book between their hands and they must keep their eyes on the book as they lift their head. Check that muscles such as splenius capitis are not overactive. Goal: 10 × 10-second holds
Muscle length test: upper trapezius, latissimus dorsi, pectoralis minor, pectoralis major, levator scapulae, anterior and middle scalenes, and the suboccipital muscles; also scored as tight or normal	Stretching of muscles determined to have decreased length Patient to perform 3 × 30-second stretches for each muscle Goal: 3 sets of 30-second holds for each muscle	Self-overpressure to stretching of muscles will be added as appropriate
Manual muscle tests performed for the lower trapezius, rhomboids, middle trapezius, and serratus anterior	Patient to perform exercises without exacerbation of symptoms Progressed based on patient response All patients begin with thin elastic bands Goal: 3 sets of 10	Patient will be progressed to medium, heavy, and extra heavy for resistance as appropriate, based on the patient’s ability

#### Exercise: 15 Minutes

Individuals randomized to both groups will receive exercise designed to improve performance of both the deep neck flexor musculature as well as the scapular musculature. The physical examination will guide exercise interventions. See the exercise intervention treatment algorithm in Table 3 [61]. Exercise will be performed after manual therapy and before dry needling. The goal of this program is to increase endurance and control of the muscles in the cervicothoracic region. The exercise portion will also include a stretching program targeting the cervicothoracic muscles, which have been placed in a shortened position as a result of poor postures. Patients will be instructed to perform the exercises as a home program twice daily. Each patient will be provided with a home exercise log that includes pictures as well as descriptions of all exercises. This exercise log will be used to encourage patient compliance.

The patient will be instructed to maintain usual activity levels within the limits of pain. Advice to maintain usual activity has been found to assist in recovery from neck pain. Patients will be instructed to do all activities that do not increase symptoms and to avoid activities that aggravate symptoms.

#### Physical Examination Techniques That Will Guide Exercise Intervention

##### Neck Flexor Endurance

Endurance of the neck flexors will be assessed with the patient lying supine in a hook-lying position. The patient will retract the chin and lift the head and neck until the head is approximately one inch above the plinth. Once in position, a line will be drawn across one of the skin folds along the patient’s neck while the therapist maintains support just under the patient’s occiput. When either the line edges begin to separate or if the patient’s head touches the therapist’s hand for more than one second, the test will be terminated. This technique has been demonstrated to have moderate reliability (ICC of .67) [62].

##### Muscle Length Assessment

Length of the upper trapezius, latissimus dorsi, pectoralis minor, pectoralis major, levator scapulae, anterior and middle scalenes, and the suboccipital muscles will be assessed according to the descriptions provided by Cleland et al [60]. Percent agreement between examiners in our previous work ranged from 77% to 85% [63].

##### Muscle Strength Assessment

Strength of the upper quadrant will be tested according to the techniques described by Kendall [64]. Percent agreement between examiners in our previous work ranged from 81% to 91% [63].

#### Dry Needling: 15 Minutes

Individuals randomized to the MTEX-Needle group will receive dry needling targeting the posterior musculature of the cervical and thoracic spine. Dry needling will occur after the manual therapy and exercise at each treatment session. The physical examination findings will guide clinicians to allow them to determine which specific muscles will be targeted (see Table 4). Examples of posterior muscles that can be treated include the trapezius, levator scapulae, splenius capitis, semispinalis, spinalis capitis, multifidi, and suboccipital muscles. The therapist will needle at least six sites up to a maximum of 10 based on identification of MTrPs. If six sites failed to be identified, the therapist will address as many sites that are present and document the number of sites treated. Once the needle has been inserted manually into the trigger point, the needle will be pistoned in an up-and-down fashion so that 2- to 3-mm vertical motions occur (ie, fast-in and fast-out technique as described by Hong) at approximately 1 Hz for 25-30 seconds, with the aim of eliciting local twitch responses [65]. The maximum number of sessions of dry needling each participant will receive is six sessions, but therapists are instructed that if the patient has complete resolution of trigger points they do not need to continue with dry needling in subsequent sessions. The therapist also may discharge a patient at the therapist’s and patient’s discretion, as would normally be done in clinical practice.

#### Physical Examination That Will Guide Dry Needling Intervention: Trigger Point Assessment

The neck and upper quarter will be examined for the presence of the following: a hypersensitive spot in a palpable taut band, palpable or visible local twitch on pincer palpation, and reproduction of referred pain elicited by palpation of the sensitive spot. These criteria have been shown to exhibit good interexaminer reliability (κ=.84-.88) when applied by an experienced clinician [66].

#### Sham Dry Needling: 15 Minutes

Park Sham acupuncture needles (AcuPrime) will be used to perform sham dry needling in all patients randomized to the MTEX-Sham group. These needles have been reported to be indistinguishable from real needles in a patient who has not experienced dry needling before [67]. The device consists of two plastic tubes that slide into one another and allow the blunted needle to cause a pricking sensation when pushed against the skin. This sham needle allows the patient to have the feeling that the needle is entering the skin while also maintaining therapist-patient contact time and treatment explanation. Sham dry needling is proposed to have less effect when compared to true dry needling [68]. Sham dry needling sites will be determined in the exact same fashion as in the MTEX-Needle group by the physical therapist after assessment. Therapists will be asked to sham needle at least six sites up to a maximum of 10, but the muscles that are (sham) treated will be left at the discretion of the physical therapist. The number of sites and specific muscles (sham) treated will be recorded by the therapist. Only posterior muscles of the cervical spine and upper thoracic spine, the same muscles targeted in the dry needling group, will be treated in order to ensure patients will be blinded to whether they received the real or sham needling.

### Data Analysis

Descriptive statistics, including frequency counts for categorical variables and measures of central tendency and dispersion for continuous variables, will be calculated to summarize the data. Baseline demographic data will be compared across treatment groups to assess the adequacy of the randomization.

**Table 4:** Dry needling intervention algorithm for treatment selection.

Assessment	Treatment
Trigger point assessment performed on the trapezius	Patient in prone, therapist identifies the hypersensitive spot in the trapezius
	The overlying skin will be cleansed with alcohol
	Once the needle has been inserted manually into the trigger point, the needle will be pistoned in an up-and-down fashion so that 2- to 3-mm vertical motions occur (ie, fast-in and fast-out technique as described by Hong) at approximately 1 Hz for 25-30 seconds, with the aim of eliciting local twitch responses
	After needle is removed, pressure with a cotton ball will be maintained to prevent excessive bleeding
	The number of sites and specific muscles treated will be recorded by the therapist
Trigger point assessment performed on the levator scapulae	Patient in prone, therapist identifies the hypersensitive spot in the levator scapulae
	The overlying skin will be cleansed with alcohol
	Once the needle has been inserted manually into the trigger point, the needle will be pistoned in an up-and-down fashion so that 2- to 3-mm vertical motions occur (ie, fast-in and fast-out technique as described by Hong) at approximately 1 Hz for 25-30 seconds, with the aim of eliciting local twitch responses
	After needle is removed, pressure with a cotton ball will be maintained to prevent excessive bleeding
	The number of sites and specific muscles treated will be recorded by the therapist
Trigger point assessment performed on the splenius capitis, semispinalis, spinalis capitis, and multifidi	Patient in prone, therapist identifies the hypersensitive spot in the splenius capitis, semispinalis, spinalis capitis, or multifidi
	The overlying skin will be cleansed with alcohol
	Once the needle has been inserted manually into the trigger point, the needle will be pistoned in an up-and-down fashion so that 2- to 3-mm vertical motions occur (ie, fast-in and fast-out technique as described by Hong) at approximately 1 Hz for 25-30 seconds, with the aim of eliciting local twitch responses
	After needle is removed, pressure with a cotton ball will be maintained to prevent excessive bleeding
	The number of sites and specific muscles treated will be recorded by the therapist
Trigger point assessment performed on the suboccipital muscles	Patient in prone, therapist identifies the hypersensitive spot in the suboccipital muscles
	The overlying skin will be cleansed with alcohol
	Once the needle has been inserted manually into the trigger point, the needle will be pistoned in an up-and-down fashion so that 2- to 3-mm vertical motions occur (ie, fast-in and fast-out technique as described by Hong) at approximately 1 Hz for 25-30 seconds, with the aim of eliciting local twitch responses
	After needle is removed, pressure with a cotton ball will be maintained to prevent excessive bleeding
	The number of sites and specific muscles treated will be recorded by the therapist

We will compare baseline variables between groups by using independent *t* tests or Mann-Whitney U tests for continuous data and chi-square tests of independence for categorical data. An intention-to-treat analysis will be utilized, in which all participants will be analyzed in the group to which they were originally assigned. All dropouts and the reasons for dropping out of the study will be reported. An a priori alpha level of .05 will be used for all analyses. All data will be checked to ensure they meet the assumptions for the inferential statistical analyses described below. If they do not meet the necessary assumptions, appropriate nonparametric procedures will be utilized. We will examine the primary aim with a two-way repeated-measures analysis of variance with treatment group (ie, manual therapy, exercise, and dry needling vs manual therapy, exercise, and sham dry needling) as the between-subjects independent variables and time (ie, baseline, 4 weeks, 6 months, and 12 months) as the within-subjects independent variable. The hypothesis of interest is the two-way group × time interaction. Bonferroni-corrected post hoc tests will be used to determine difference between group means.

### Power Analysis

Sample size and power calculations were performed using G*Power version 2 statistical software (Heinrich-Heine- Universität Düsseldorf) based on the minimal clinical improvement of 12 points on the NDI [51,69], assuming a standard deviation of 16 points, two-tailed, and an alpha level of .05. This requires a minimum sample size of 29 subjects per group. A total of 76 patients with a primary complaint of neck pain who meet the inclusion/exclusion criteria and consent to participate will be enrolled into the study. This sample size will yield greater than 80% power to detect both statistically significant and clinically meaningful changes in the NDI; additionally, this will control for dropouts prior to the 4-week follow-up.

### Risks

The risks associated with a patient’s participation in this study are minimal. Patients may experience an increase in pain intensity from completing the range of motion exercise due to a muscle or ligament injury. Based on our clinical experience, the chance of this happening is rare, which means it occurs in less than 1% of people. We have attempted to minimize this risk by having a licensed physical therapist examine all patients and instruct them in the proper exercise technique. In addition, a therapist will re-examine a patient at any time, if appropriate. It is also possible that patients will experience mild muscle soreness after the manipulation is performed. Based on our clinical experience, the chance of this happening is common, which means it occurs in 1%-25% of people. However, this soreness typically resolves within 1-48 hours after manipulation. We have minimized the risks associated with manipulation by ensuring that the licensed physical therapists participating in this study already routinely use manipulation in the management of patients with neck pain. We have further minimized this risk by ensuring that each physical therapist participating in this study has been specifically trained in the use of the manipulation techniques to be used in this study. Furthermore, all potential subjects will be screened to ensure they do not exhibit any exclusion criteria that may place the individual at increased risk for a serious complication.

When dry needling treatment is performed, it is possible that patients will experience the following common adverse events: bruising, bleeding, pain during treatment, pain after treatment, or aggravation of symptoms 1.7%-7.6% of the time (~2-8 out of 100). Uncommon adverse events include the following: drowsiness, headache, or nausea 0.13%-0.26% of the time (~1-3 out of 1000). Possible rare adverse events include fatigue, altered emotions, shaking, itching, claustrophobia, or numbness 0.01%-0.04% of the time (1-4 out of 10,000). Dry needling is very safe; however, the most serious side effect from dry needling is pneumothorax (ie, lung collapse due to air inside the chest wall), which can occur in less than 0.01% (<1 out of 10,000) treatments. This risk is very low and in a recent survey of physical therapists who use dry needling, Brady et al reported that no episodes of pneumothorax occurred in over 7600 treatments. We have minimized the risks associated with dry needling by ensuring that the licensed physical therapists participating in this study already routinely use dry needling in the management of patients with neck pain. We have further minimized this risk by ensuring that each physical therapist participating in this study has been specifically trained in the use of the dry needling techniques to be used in this study. Furthermore, all potential subjects will be screened to ensure they do not exhibit any exclusion criteria that may place the individual at increased risk for a serious complication.

Should any adverse event occur, it would be appropriately managed by the treating physical therapist by activating emergency services if immediate medical attention is required; standard clinical advice will be used in the case of minor events, such as transient soreness.

## Results

This trial is registered at ClinicalTrials.gov (NCT02731014) and recruitment is currently underway and is expected to be completed by the end of 2017. Data collection for long-term outcomes will occur throughout 2017 and 2018. Data analysis, preparation, and publication submission is expected to occur throughout the final three quarters of 2018. See Table 5 for the study timeline and milestones.

**Table 5:** Timeline and milestones.

Activity	2016 (quarter)		2017 (quarter)				2018 (quarter)			
	3	4	1	2	3	4	1	2	3	4
Participant recruitment	X	X	X	X	X	X				
Data collection for long-term outcomes			X	X	X	X	X	X	X	X
Data analysis, preparation, and submission for publication								X	X	X
Publication submissions										X

## Discussion

### Principle Findings

Neck pain is commonly unresponsive or does not fully resolve with current treatment strategies, with 37% of patients going on to experience chronic neck pain of greater than one year [2]. Dry needling may be one intervention that could lead to improved outcomes when used in conjunction with exercise and manual therapy [11,39]. Anecdotally, in the clinical setting, a patient’s pain level commonly limits their ability to participate in active exercise interventions. As dry needling appears to have a significant treatment effect on reducing pain and increasing pressure pain threshold [38], it may facilitate a patient’s ability to perform a prescribed exercise program. In addition, it may improve patient compliance with exercise, which may lead to improved results from an exercise program.

Therefore, the aim of this trial is to determine if the addition of dry needling to an exercise and manual therapy treatment program will further reduce pain and improve disability in patients with mechanical neck pain, as compared to exercise and manual therapy and sham needling.

We hypothesize that patients who receive dry needling, manual therapy, and exercise will achieve greater reductions in pain and disability in the short term (ie, 4 weeks) and long term (ie, 6 and 12 months) compared to those who receive sham dry needling, manual therapy, and exercise.

As the use of dry needling by physical therapists becomes more widespread, and more therapists are trained in this approach, research is needed to support or refute its effectiveness. The results of this trial will assist in providing long-term outcomes examining the effectiveness of dry needling, which are currently lacking in the literature.

We anticipate the potential challenges to this study to include the following: difficulty with patient recruitment, patient compliance with follow-up schedule, and patients lost to follow-up over the 1-year, long-term, follow-up period. To address these challenges, we have utilized two large clinics in different locations in the United States to improve the ability to recruit patients in a timely manner. Further, we will provide a small financial reimbursement for patients as incentive for completion of the 4-week, 6-month, and 12-month follow-up in order to assist with compliance and reduce the numbers lost to follow-up.

### Limitations

We recognize that there are a number of potential limitations in the study design. The treating therapists cannot be blinded to group assignment, which may influence the verbal and nonverbal interaction with subjects. To try to manage this limitation, all therapists will be trained to maximize the consistency with which the dry needling intervention and the sham intervention will be performed.

We have chosen to allow therapists to perform individualized dry needling treatment specific to each patient within the outlined treatment algorithm to be consistent with clinical practice and improve external generalizability. We believe that providing treatment specific to the patient’s presentation will improve outcomes. We understand this may be seen as a limitation as it may lead to variation in the treatments that will be applied by therapists, which may mask the difference between groups. However, individualized treatment better reflects clinical practice.

Another potential limitation is that we are not including physical measures such as range of motion or pain pressure threshold in our analyses. We have chosen to limit our outcomes to validated questionnaires in an effort to decrease loss to follow-up, especially at the long-term time points.

## Appendix

Multimedia Appendix 1 APTA Grant review peer review report.

Multimedia Appendix 2 CONSORT‐EHEALTH checklist (V.1.6.1).

## Abbreviations

GROC: Global Rating of Change Scale
ICC: inter-class correlation
MTEX-Needle: manual therapy, exercise, and dry needling
MTEX-Sham: manual therapy, exercise, and sham dry needling
MTrP: myofascial trigger point
NDI: Neck Disability Index
VAS: Visual Analog Scale
