# Supplementary material for: Dry Needling for Patients With Neck Pain: Protocol of a Randomized Clinical Trial
Source: JMIR Res Protoc. 2017 Nov 22;6(11):e227. doi: 10.2196/resprot.7980 (PMC5719229; doi:10.2196/resprot.7980)
Supplement: Multimedia Appendix 1 [file resprot_v6i11e227_app1.pdf]

Orthopaedic Section of the APTA Grant Review

PI Name: Eric Gattie
Title: Dry Needling for Patients with Neck Pain: A Randomized Controlled Trial
Grant Category: New Investigator

SCORES: 225, 250, 150

Pre-CSM AVERAGE SCORE: 208.3

Scoring guidelines
100-150: Outstanding, should be funded essentially as is
150-200: Excellent, potentially to be funded with minor changes
200-250: Very Good, potentially to be funded with major changes
250-350: Good, low potential to be funded even with changes
350-500: Acceptable, not likely to be funded

SUMMARY:

To be completed after External Reviewers Meeting

- Optimal dose is not clear and how far should outcomes be examined.
- Multiple treatments in addition to dry needling.
- Can they recruit the 76 subjects and 1-year follow-up? Can it be done in 2 years?
- Powered on the short-term outcomes but focusing on long-term outcomes.
- Strong environment and co-I's
- Why is it single blind? Why could the PT not be blinded? Person collecting data should be blind. However it is clinically translatable.
- Limited control over how the intervention will be delivered.
- Will everyone have a trigger point? What if they don't have a trigger point?
- **No biostatistician on the project. (Must do?)**
- Team is strong; balance of clinician and researcher
- Preliminary work might be needed.
- Tighten down inclusion criteria. What about those who don't want dry needling. The needling may not be completely pragmatic and may be overdosing the needling.

Must do for Funding:

- **Add biostatistician**
- **Must show evidence that this can be done in 2 years max.**

To be completed if recommended for Funding

Primary Reviewer

1. **Significance**

Neck pain is a common problem and current treatment approaches are not optimal. Identifying better treatment strategies would be beneficial to outpatient PTs. The authors advocate for the need for a trial with "realistic treatment time frames and methods consistent with clinical practice"; however, not clear the parameters of these are established

2. **Approach**

Purpose indicated as to examine the effectiveness of dry needling; however, really considering whether the addition of dry needling to a program of exercise and manual therapy is more effective than exercise and manual therapy alone

The authors suggest clinicians commonly perform an examination to locate MTrPs and needle numerous active TPs in a single session and typically will see patients over 5-7 treatments to "achieve optimal effects"; however, are standard practice patterns of dry needling known and have dose- response studies been performed. My concern is, should this study be funded and the results not support dry needling, another group will simply discount the results and suggest optimal results would have occurred with a different approach or dosage.

The authors cite a study they have previously done comparing dry needling to trigger point manual therapy over two treatment sessions with null findings beyond changes in pressure pain threshold favoring the dry needling group. Not clear how this study with both groups receiving manual therapy is expected to find differences in the primary outcome of NDI over a longer period. The choice of comparison seems designed for failure.

The authors anticipate successfully recruiting 76 patients from their clinics in < 2 years; however, with the 1 year follow up time, will they be able to complete the study within the 2 year period of the grant? The timeline and milestone figure indicates recruitment through 2016 with data collection and longer term outcomes only through half of 2017. Unless, all participants are recruited within the first half of 2016, 1 year follow up is not possible

The manual therapy algorithm is dependent upon the finding of "hypomobility". What if a participant is not found to have "hypomobility" in the cervical or thoracic spine? Similarly, for the dry needling, what if trigger points are not observed in individual participants?

Not clear why the study is powered on a 4 week change in NDI when the authors continually suggest a significant limitation in the literature is the need for studies with longer follow up

Patient costs are specified as completed at 30 months; however, study timeline indicates 2 years

3. **Innovation**

The study is mildly innovative in considering 1 year outcomes

The authors indicate "our recent systematic review"; however, do not provide a citation. Has this been published? Regardless, they indicate their review observed a lack of pragmatic methodologies consistent with the common application in clinical practice. Not clear to me that common application in clinical practice is known. In my opinion, this proposal would be more innovative if considering dose response question with short term follow up rather than the current design focused on 1 year outcome

4. **Investigative Team**

5. **Environment / Facilities**
   An strong investigative team and environment suggesting the ability to complete the study and disseminate the findings

Significance 200
Approach 250
Innovation 250
Investigative Team 150
Environment / Facilities 150

225

Secondary Reviewer #1

1. **Significance**
   Strengths
   - Dry needling is a "hot topic" but well designed clinical trials are lacking to demonstrate it's effectiveness
   - Long term effectiveness has not been established in the few previous investigations of dry needling
   - A two-session RTC showed improvements in pain pressure threshold (compared to manual therapy of trigger points)

   Weakness
   - Preliminary work on responders vs nonresponders to dry needling with mechanical neck pain has not been established
   - Highly speculative ideas on mechanisms that dry needling acts on (e.g. CNS)

2. **Approach**
   Strengths
   - Patients in both groups receive equal time and attention
   - Questions about dry needling are specifically asked – unsure what rating scale will be used to determine that the sham was successful or not.
   - Outcomes are assessed at 4 weeks, 6 months, and 1 year
   - 8 PTs at 7 clinics will improve external validity

   Weakness

- The two specific aims seem to be saying the same thing and there is only 1 hypothesis. How will the information advance our understanding of who it works for, what works, and why dry needling works?
- Surveys ask a number of questions about patient expectation but there is no data analysis plan. Would make a good aim if this were incorporated.
- "Well defined sample" but there is age range and inclusion criteria are quite broad – why not limit to people of a certain age (e.g. younger to limit potential for cervical degenerative disc disease). Sample is not large enough to stratify by age. Unclear how applicable the expected effect size and variability used in the power analysis are to this diagnosis group and intervention.
- single blind trial introduces potential for bias – why can't the PT be blinded to the dry needling intervention? Why can't the person collecting data be blinded to the subject's group assignment?
- multi-modal care = manual therapy, exercise, and dry needling. Evidence has not yet clearly defined the parameters for manual therapy and exercise – only that it is better than wait listing and stand alone intervention. Now another intervention is added. How will this advance our understanding of the factors and effective dosage for success?
- What is the justification for 7 visits over 4 weeks? Is this what has been found to be effective for manual therapy and exercise? Or, is this based on insurance norms?
- Mechanisms of dry needling are not well understood and information about local and CNS changes are highly speculative
- Lacks a biostatistician to run more sophisticated analyses to 1) Evaluate the patients who are lost to follow-up compared to those who complete the follow-up assessments; 2) Adjust for the baseline measurement of the outcome variable, comorbidities, and other relevant factors; 3) Impute missing variables in the intent-to-treat analysis with a sub-model that uses available covariates to account for plausible patterns of non-random missing data
- Lacks a plan for assessing PT compliance with protocols
- Anticipating 6 months to get IRB approval making it a 2.5 year study

3. **Innovation**
   - Dry needling is a relatively new intervention. There are a lack of well designed, clinical studies to test its efficacy.
   - Use of a placebo needle set up is a strength.

4. **Investigative Team**

   Dr. Gattie is the PI – this is his dissertation research from the University of Newcastle, Australia. Has experience with dry needling since completing a manual therapy fellowship in Colorado. Is employed in the Concord Hospital system where the research will occur. Also is an adjunct at Franklin University. Has presented case reports at national conferences using dry needling as an intervention. Has not yet published in peer-reviewed literature. No other funding support.

Dr. Snodgrass is a Co-I and the dissertation advisor. She has a strong publication history in more mechanistic work regarding cervical pain and stiffness. Experience with randomized clinical trials appears limited.

Josh Cleland is a Co-I. He has conducted a number of phase 1-2 clinical trials at Concord Hospital. He has recent experience with investigations of dry needling. He is a prolific author. His major funding sources have been from APTA.

Lack of a biostatistician is a major limitation.

5. **Environment / Facilities**
   - Dr. Cleland has led a number of small scale RTCs at Concord Hospital System
   - Letters of support indicate that the healthcare managers are supportive of the trial

250

Secondary Reviewer #2

1. **Significance**

Strengths
- Neck pain is common, costly and has a high frequency of recurrence despite existing treatments.
- Highly prevalent population in outpatient physical therapy setting
- Recent Cochrane reviews have highlighted the lack of quality evidence to support physical therapy modalities and treatments.

Weaknesses
- None noted

2. **Approach**

Strengths
- RCT with placebo control.
- Placebo needles have been validated and tested against the experimental condition.
- Inclusion and exclusion criteria are well described.
- The treatment interventions are well described and are stringent enough to allow for a controlled comparison, but flexible enough to allow for best practice and individualized patient care.
- Recruitment will be from a consecutive pool of patients, which may reduce the selection bias

Weaknesses

- Treatment will be performed across seven clinics, which may increase the heterogeneity of treatment delivery.

3. **Innovation**

Strengths
- This study will overcome previous limitations in the field by using dry needling in combination with a usual rehabilitation program that consists of other intervention components, and will include a longer-term outcome.
- High quality evidence evaluating this newer intervention in the field is currently lacking.

Weaknesses
- None noted

4. **Investigative Team**

Strengths
- Supportive and well-published mentors in the area of neck pain and clinical trials.
- The research team is well rounded to include a balance of clinical and research expertise.

Weaknesses
- The physical location of the Co-I may impede progress, although the PI has outline a plan for communication with his mentors and collaborators

5. **Environment / Facilities**

Strengths
- PI reports that 270 patients with neck pain are seen in the clinics on an annual basis, which will support their recruiting efforts.
- Appropriate for all components of the study.

Weaknesses
- None noted

Significance: 150
Approach: 125
Innovation: 150
Investigative Team: 150
Environment / Facilities: 200

150
